# Outbreak of Epidemic Keratoconjunctivitis Caused by Human Adenovirus Type D53 in an Eye Care Clinic — Los Angeles County, 2017

**DOI:** 10.15585/mmwr.mm6748a4

**Published:** 2018-12-07

**Authors:** Kelsey OYong, Marie Killerby, Chao-Yang Pan, Thalia Huynh, Nicole M. Green, Debra A. Wadford, Dawn Terashita

**Affiliations:** ^1^Acute Communicable Disease Control Program, Los Angeles County Department of Public Health, Los Angeles, California; ^2^National Center for Immunization and Respiratory Diseases, Division of Viral Diseases, CDC; ^3^Viral and Rickettsial Disease Laboratory, California Department of Public Health, Richmond, California; ^4^Public Health Laboratory, Los Angeles County Department of Public Health, Los Angeles, California.

## Abstract

On June 22, 2017, the Los Angeles County Department of Public Health (LAC DPH) was notified of seven patients who were seen at an eye care clinic on June 8, 2017, and later developed symptoms of epidemic keratoconjunctivitis (EKC). EKC is a contagious, severe form of viral conjunctivitis that can cause pain and blurred vision for up to 4 weeks (*1*). LAC DPH conducted an investigation, which identified 17 patients with EKC, including 15 who had visited the optometry clinic and two who were household contacts of clinic patients. Observations in the clinic found deficiencies in disinfection of tonometers (an instrument connected to a slit lamp and used to test for glaucoma by measuring intraocular pressure) and multiuse eye drop administration. Staff member education and revision of disinfection practices interrupted further transmission. Patient specimens tested positive for human adenovirus (HAdV) type D53 (HAdV-53). As the first documented EKC outbreak associated with HAdV-D53 in the United States, this outbreak highlights the need for rigorous implementation of recommended infection prevention practices in eye care settings.

## Investigation and Results

On June 22, 2017, hospital A reported a cluster of seven patients with EKC who had been seen at an affiliated optometry clinic to LAC DPH. Staff members who provide care at the clinic include three optometrists, one ophthalmologist, and three optometric assistants. The clinic has three exam rooms and sees an average of 1,300 patients each month. LAC DPH subsequently began an investigation into the cluster.

A case was defined as 1) diagnosis of EKC, adenoviral conjunctivitis, or viral conjunctivitis by an ophthalmologist or optometrist; or 2) laboratory confirmation of HAdV from a specimen collected by conjunctival swab in a person seen at the optometry clinic during June 5–July 3, 2017. A health care–linked case was defined as a case of EKC in a person who had visited the optometry clinic during June 7–July 3, 2017, and had symptom onset within 21 days of their visit. A household case was defined as an EKC case in a household or family contact of a patient with EKC.

All patients with EKC were symptomatic and self-referred to a health care provider. Review of optometry clinic medical records and telephone calls to patients did not identify additional cases. Among the 17 patients with EKC, 15 met the health care–linked case definition, including patient A, who appeared to be the source of introduction into the clinic (Figure). Two additional patients met the household case definition; both reported that a spouse was symptomatic before their own illness onset.

**FIGURE:**
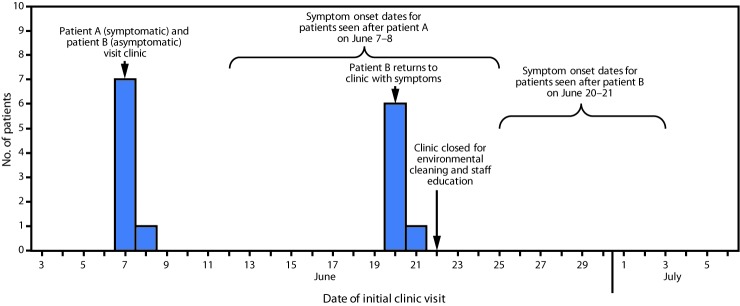
Health care–linked cases of epidemic keratoconjunctivitis (N = 15), by date of initial eye care clinic visit — Los Angeles County, California, June–July 2017

The median patient age was 62 years (range = 43–78 years), and 12 patients were women. No hospitalizations resulted from infection, although seven patients had more than one visit to a clinic, a hospital emergency department, or an urgent care center for symptoms. Patients had symptoms consistent with EKC, including eye redness (14) and discharge (13). The mean incubation period was 9 days (range = 5–19 days).

Review of health care–linked patient clinic visit dates preceding symptom onset revealed two apparent clusters. Patient A visited the clinic on June 7 with symptoms consistent with EKC, before the initial visits of seven additional patients on June 7 and June 8; these patients’ EKC symptoms began during June 12–25. On June 20, a patient who went to the clinic on June 7 (patient B) returned to the clinic with EKC symptoms that had begun on June 14. Another seven patients visited the clinic after patient B on June 20 and June 21, before the onset of their EKC symptoms (June 26–July 3), consistent with transmission to these additional seven patients.

Medical chart review indicated common exposures among the 14 health care–linked patients: all were examined by the same optometrist in the same exam room after either patient A (June 7) or patient B (June 20) had been seen. No health care personnel reported EKC symptoms before or during the outbreak period. Among the 14 patients, other exposures included slit lamp contact (13), tonometry (12), and receipt of dilating eye drops from a multidose container (10). Use of multidose sodium fluorescein eye drops was reported for six patients in the first cluster and none in the second. During patient A’s initial clinic visit on June 7, sodium fluorescein drops from a multiuse vial were administered, and a slit lamp examination was performed.

The clinic closed on June 22 for intensive environmental cleaning of clinic surfaces and equipment, instrument cleaning and disinfection, and to provide training to staff members on infection prevention. The clinic reopened the following day.

On June 23, LAC DPH conducted an announced site visit to inspect the premises, observe infection prevention practices, interview staff members, and review infection prevention policies. Clinic patients typically proceed from the waiting area to one of three exam rooms, each with its own slit lamp with tonometer. Observations and staff member interviews indicated gaps in infection prevention practices, including use of eye drops from multidose vials on multiple patients, occasionally touching the eye or surrounding area, and reprocessing of tonometers using a 70% isopropyl alcohol wipe rather than the recommended 5–10-minute disinfecting soak with chlorine or ethyl alcohol.*

Conjunctival swab specimens from four symptomatic patients were sent to the LAC Public Health Laboratory for conventional and shell vial culture (used for adenovirus detection) (*2*) and detection by fluorescent monoclonal antibody staining; adenovirus was detected in two specimens. Specimens from an additional 11 patients were tested at the laboratory of hospital A, and adenovirus was identified in six by viral culture.

Specimens from the eight patients with positive adenovirus cultures were then submitted to the California Department of Public Health Viral and Rickettsial Disease Laboratory (VRDL) for HAdV detection and molecular typing by sequence analysis of the hypervariable region of the HAdV hexon gene and the HAdV group-specific region of the fiber gene (*3*,*4*). All eight patient specimens were positive for HAdV-D53. Subsequently, VRDL generated HAdV-D53 whole genome sequences from one patient specimen, which was nearly identical to a recently reported whole genome sequence of HAdV-D53 from Japan (GenBank sequence LC215428).

## Discussion

HAdV-D53 has been recognized as an agent of EKC outbreaks in Japan since 1980 (*5*–*7*) and in Germany since 2005 (*8*). However, HAdV-D53 has not previously been reported to the U.S. National Adenovirus Type Reporting System, and this is the first reported EKC outbreak associated with HAdV-D53 in the United States.

Based on this investigation, it is believed that the virus was introduced to surfaces in the exam room by a symptomatic patient, and that subsequent lapses in infection prevention practices led to transmission to other patients. Previous studies have demonstrated that adenoviruses can persist on environmental surfaces for several weeks (*9*). Enhanced infection prevention practices, including staff member education on eye drop administration and longer slit lamp and tonometer disinfection times were implemented. No further cases were reported after July 3, 2017.

Previous similar EKC outbreaks have been linked to eye care clinics employing improper disinfection practices and lapses in hygienic protocols (*10*). To prevent EKC transmission in eye care settings, recommended practices include the use of disposable tonometer tips, disinfectants efficacious against adenoviruses for tonometers and slit lamps, and single-use eye drops when available. Use of recommended infection prevention practices is necessary to avoid EKC and other health care–associated infections.

**Summary**

**What is already known about this topic?**

Epidemic keratoconjunctivitis (EKC) associated with adenovirus is a frequent cause of outbreaks in eye care settings. Previous outbreaks have been associated with lapses in infection prevention.

**What is added by this report?**

This report details the first documented outbreak of adenovirus D53 EKC in the United States. Seventeen EKC cases were identified; after the primary case, all cases occurred in eye care clinic patients or their household contacts. Infection prevention lapses were associated with the outbreak, specifically improper ocular equipment disinfection.

**What are the implications for public health practice?**

By understanding the associated causes for transmission, health care practitioners and public health officials can target resources to ensure proper infection prevention practice.

## Footnotes

* https://www.cdc.gov/infectioncontrol/pdf/guidelines/disinfection-guidelines.pdf.

## References

[1] Lu X, Joshi A, Flomenberg P. Adenoviruses. In: Kaslow RA, Stanberry LR, LeDuc JW, eds. Viral infections of humans. New York, NY: Springer; 2014:99–121.

[2] Espy MJ, Hierholzer JC, Smith TF. The effect of centrifugation on the rapid detection of adenovirus in shell vials. Am J Clin Pathol 1987;88:358–60. 10.1093/ajcp/88.3.358 3307377

[3] McCarthy T, Lebeck MG, Capuano AW, Schnurr DP, Gray GC. Molecular typing of clinical adenovirus specimens by an algorithm which permits detection of adenovirus coinfections and intermediate adenovirus strains. J Clin Virol 2009;46:80–4. 10.1016/j.jcv.2009.06.008 19577957 PMC2846087

[4] Lu X, Erdman DD. Molecular typing of human adenoviruses by PCR and sequencing of a partial region of the hexon gene. Arch Virol 2006;151:1587–602. 10.1007/s00705-005-0722-7 16502282

[5] Engelmann I, Madisch I, Pommer H, Heim A. An outbreak of epidemic keratoconjunctivitis caused by a new intermediate adenovirus 22/H8 identified by molecular typing. Clin Infect Dis 2006;43:e64–6. 10.1086/507533 16941356

[6] Aoki K, Ishiko H, Konno T, Epidemic keratoconjunctivitis due to the novel hexon-chimeric-intermediate 22,37/H8 human adenovirus. J Clin Microbiol 2008;46:3259–69. 10.1128/JCM.02354-07 18701656 PMC2566102

[7] Kaneko H, Aoki K, Ishida S, Recombination analysis of intermediate human adenovirus type 53 in Japan by complete genome sequence. J Gen Virol 2011;92:1251–9. 10.1099/vir.0.030361-0 21402595

[8] Binder AM, Biggs HM, Haynes AK, Human adenovirus surveillance—United States, 2003–2016. MMWR Morb Mortal Wkly Rep 2017;66:1039–42. 10.15585/mmwr.mm6639a2 28981484 PMC5720882

[9] Killerby ME, Stuckey MJ, Guendel I, Notes from the field: epidemic keratoconjunctivitis outbreak associated with human adenovirus type 8—U.S. Virgin Islands, June–November 2016. MMWR Morb Mortal Wkly Rep 2017;66:811–2. 10.15585/mmwr.mm6630a3 28771460 PMC5720879

[10] King D, Johnson B, Miller D, Adenovirus-associated epidemic keratoconjunctivitis outbreaks—four states, 2008–2010. MMWR Morb Mortal Wkly Rep 2013;62:637–41. 23945769 PMC4604776

